# Engineering Carbon Nitride Quantum Dots via Sulfur Doping for Controlled Reactive Oxygen Species Generation

**DOI:** 10.1002/gch2.202500447

**Published:** 2026-01-08

**Authors:** Nikita Belko, Hanna Maltanava, Nadzeya Brezhneva, Konstantin Tamarov, Vesa‐Pekka Lehto, Jani O. Moilanen, Jari T.T. Leskinen, Dmitry Semenov, Elena Filonenko, Igor Koshevoy, Jacob Schneidewind, Winnok H. De Vos, Polina Kuzhir

**Affiliations:** ^1^ Department of Physics and Mathematics University of Eastern Finland Joensuu Finland; ^2^ Institute of Organic Chemistry and Macromolecular Chemistry Friedrich Schiller University Jena Jena Germany; ^3^ Center for Energy and Environmental Chemistry Jena (CEEC Jena) Friedrich Schiller University Jena Jena Germany; ^4^ Department of Technical Physics University of Eastern Finland Kuopio Finland; ^5^ Department of Chemistry, Nanoscience Centre University of Jyväkyla Jyväkylä Finland; ^6^ School of Computing University of Eastern Finland Joensuu Finland; ^7^ Department of Chemistry and Sustainable Technology University of Eastern Finland Joensuu Finland; ^8^ Helmholtz Institute for Polymers in Energy Applications Jena (HIPOLE Jena) Jena Germany; ^9^ Laboratory of Cell Biology and Histology, Dept. Veterinary Sciences University of Antwerp Antwerp Belgium

**Keywords:** carbon nitride quantum dots, hydroxyl radical, reactive oxygen species, singlet oxygen, superoxide

## Abstract

Carbon nitride quantum dots (CNQDs) are emerging as versatile photocatalytic materials with promising applications in biomedicine and environmental remediation. In this study, we synthesized pristine and sulfur‐doped CNQDs via a hydrothermal method, and characterized them using transmission electron microscopy (TEM), X‐ray photoelectron spectroscopy (XPS), and UV–vis absorption spectroscopy. For the first time, the quantum yields of superoxide and singlet oxygen generation were measured for CNQDs. Sulfur doping was found to significantly enhance superoxide generation while concurrently suppressing singlet oxygen production, offering a powerful mechanism for tailoring reactive oxygen species (ROS) output. In addition, all CNQD samples produced hydrogen peroxide and hydroxyl radicals. The ability of these nanomaterials to produce multiple ROS types underscores their potential as hypoxia‐resistant photosensitizers (PSs) for photodynamic therapy (PDT) and as efficient photocatalysts for pollutant degradation.

## Graphical abstract

Pristine and sulfur‐doped carbon nitride quantum dots are prepared via hydrothermal “bottom‐up” synthesis. Quantum yields of superoxide and singlet oxygen generation are measured. Sulfur doping is shown to boost photocatalytic production of superoxide by carbon nitride quantum dots while suppressing singlet oxygen generation. The quantum dots also demonstrate efficient photocatalytic hydrogen peroxide and hydroxyl radical production.

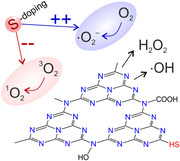

## 1 Introduction

CNQDs have garnered significant interest as nanomaterials for biomedical applications, owing to their small size, biocompatibility, excellent water dispersibility, and bright fluorescence [1, 2, 3, 4, 5, 6]. CNQDs have been successfully applied in biomedicine, particularly in bioimaging and biosensing for detecting metal ions, various bioanalytes, etc. [2, 6, 7, 8, 9, 10, 11, 12]. One of the least explored yet promising biomedical applications of CNQDs is PDT [3, 13, 14].

PDT is an emerging cancer treatment modality [15, 16, 17, 18, 19, 20, 21], offering advantages such as minimal invasiveness and high selectivity. It relies on a PS, a therapeutic agent that preferentially accumulates in cancerous tissues and generates cytotoxic species upon light activation. Most PSs require ground‐state molecular oxygen in the target tissue. Upon photoexcitation, PSs produce reactive intermediates via two primary mechanisms [22, 23]. In the Type I mechanism, electron or hydrogen atom transfer from the excited PS leads to the formation of radicals or radical ions, including superoxide radical anion ($O_{2}^{\cdot -}$) and hydroxyl radical (${\mathrm{OH}}^{.}$). In the Type II mechanism, energy transfer from the PS to ground‐state, triplet oxygen (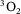) results in singlet oxygen (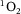) generation. These reactive species exert potent cytotoxic effects, ultimately causing cell damage and death.

A major limitation of PDT is its reduced efficacy under hypoxic conditions [24], which are commonly found in the tumor microenvironment [25, 26]. Although both Type I and Type II photoreactions depend on molecular oxygen [23], Type I PSs are generally less sensitive to oxygen concentration [27, 28, 29, 30], and molecular oxygen can be partially recycled during Type I processes [29]. Nevertheless, most PSs reported to date operate via the Type II mechanism [31]. The growing recognition of the need for hypoxia‐resistant PSs has spurred interest in developing new Type I PSs [28, 29, 30, 32]. Several recent studies have demonstrated Type I PSs capable of generating superoxide and maintaining photodynamic activity under hypoxia [32, 33, 34, 35, 36, 37, 38, 39]. In addition to superoxide, some of these PSs also produce hydroxyl radical [34, 39] and singlet oxygen [37].

In addition to biomedical applications, the generation of ROS plays a crucial role in environmental photocatalysis. ROS can be utilized for the degradation of organic pollutants, water disinfection, and industrial wastewater treatment [40], thereby contributing to sustainable environmental remediation.

Quantum yields of singlet oxygen generation ($\Phi {(}^{1}O_{2})$) have been reported for a wide range of PSs [31, 41, 42, 43, 44, 45, 46, 47, 48, 49, 50, 51]. In contrast, data on quantum yields of superoxide generation ($\Phi (O_{2}^{\cdot -})$) remain scarce. To date, $\Phi (O_{2}^{\cdot -})$ has only been measured for a few water‐soluble quinones [52], coenzymes NADH and NADPH [53], and organic matter from wastewater [54]. As a single numerical value, $\Phi (O_{2}^{\cdot -})$ is particularly useful for comparing the efficiency of different PSs. However, the range of $\Phi (O_{2}^{\cdot -})$ values for Type I PSs remains largely unexplored.

Graphitic carbon nitride (*g*‐$C_{3}N_{4}$), the bulk counterpart of CNQDs, is a well‐known photocatalyst capable of generating various ROS, including 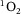 [55, 56], $O_{2}^{\cdot -}$ [57, 58, 59], $H_{2}O_{2}$ [59, 60], and ${\mathrm{OH}}^{.}$ [61]. Colloidal crystalline *g*‐$C_{3}N_{4}$ has been shown to produce 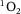, $O_{2}^{\cdot -}$, and $H_{2}O_{2}$ [62]. Moreover, $C_{3}N_{4}/{\mathrm{MnO}}_{2}$ nanoparticles have demonstrated the ability to generate both 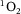 and ${\mathrm{OH}}^{.}$, enabling photodynamic effects under both normoxic and hypoxic conditions [63].

Similarly, CNQDs are expected to produce ROS upon photoexcitation. Indeed, several studies have reported ROS generation by CNQDs, although the specific types of ROS were not identified [4, 13, 14, 64]. Photocatalytic $H_{2}O_{2}$ production by CNQDs has been demonstrated [65], and both 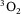 and 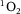 generation have been observed and utilized for corneal cross‐linking [5]. As anticipated, photoinduced ROS production by CNQDs has led to cytotoxic effects [3, 4], positioning CNQDs as promising PS candidates for PDT.

To date, ROS production by CNQDs has only been assessed qualitatively, and neither $\Phi (O_{2}^{\cdot -})$ nor $\Phi {(}^{1}O_{2})$ have been quantified for these nanomaterials. Furthermore, the impact of synthetic methods and heteroatom doping on ROS generation remains unexplored. One potential strategy to enhance ROS production is sulfur doping. Sulfur substitution has been employed in molecular PSs, resulting in increased ROS yields [20, 66]. Sulfur‐doped CNQDs have been synthesized via various methods [57, 67, 68, 69, 70, 71, 72], without compromising their biocompatibility [67, 69]. However, a direct comparison between pristine and sulfur‐doped CNQDs, particularly regarding their ROS‐generating capabilities, has not yet been conducted.

In this study, we synthesize pristine and sulfur‐doped CNQDs via bottom‐up hydrothermal methods. The prepared materials are characterized using TEM, XPS, and UV–vis absorption spectroscopy. Then, we investigate how the synthetic route and sulfur content influence the ability of CNQDs to generate various ROS, namely superoxide radical anion, hydrogen peroxide, singlet oxygen, and hydroxyl radical. For the first time, we determine quantum yields of $O_{2}^{\cdot -}$ and 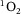 production for CNQDs.

## 2 Results and Discussion

Pristine CNQDs were synthesized from urea and citric acid, following the procedure described in ref.  [73] with modifications. This sample is hereafter referred to as CNQD‐U. Sulfur‐doped CNQDs were obtained by substituting half or all of the urea with an equimolar amount of thiourea. The sample prepared using both urea and thiourea is denoted as CNQD‐UT, while the sample prepared solely from thiourea is denoted as CNQD‐T. A more detailed description of the synthetic route is provided in Section 4.

**FIGURE 1:**
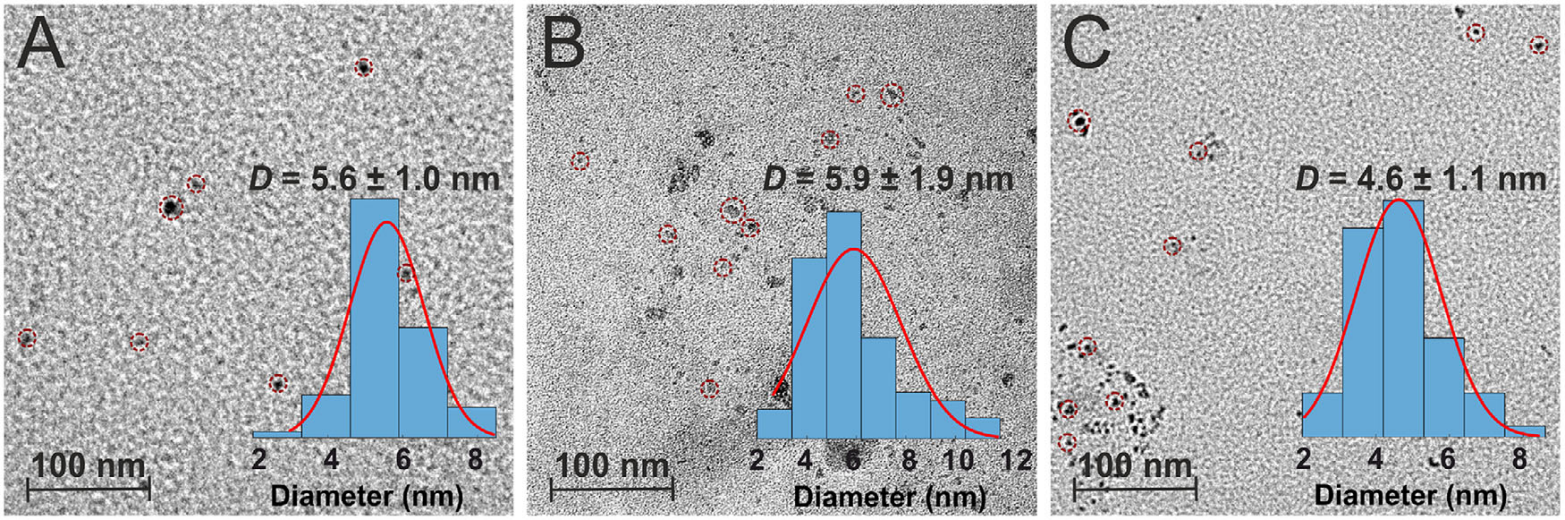
TEM images for CNQD‐U (A), CNQD‐UT (B), and CNQD‐T (C). Some of the particles are highlighted with circles. The insets show the respective particle size distributions.

TEM images (Figure 1) confirm that the synthesized materials are nanoparticles with average diameters ranging from 5 to 6 nm and moderate size dispersity. This morphology is characteristic of CNQDs and consistent with previous reports [1, 72, 74]. Sulfur doping via thiourea did not significantly alter the size of the CNQDs. The colloidal solutions of pristine and sulfur‐doped CNQDs exhibited yellow and brown coloration, respectively. All samples formed homogeneous phases without precipitation.

**FIGURE 2:**
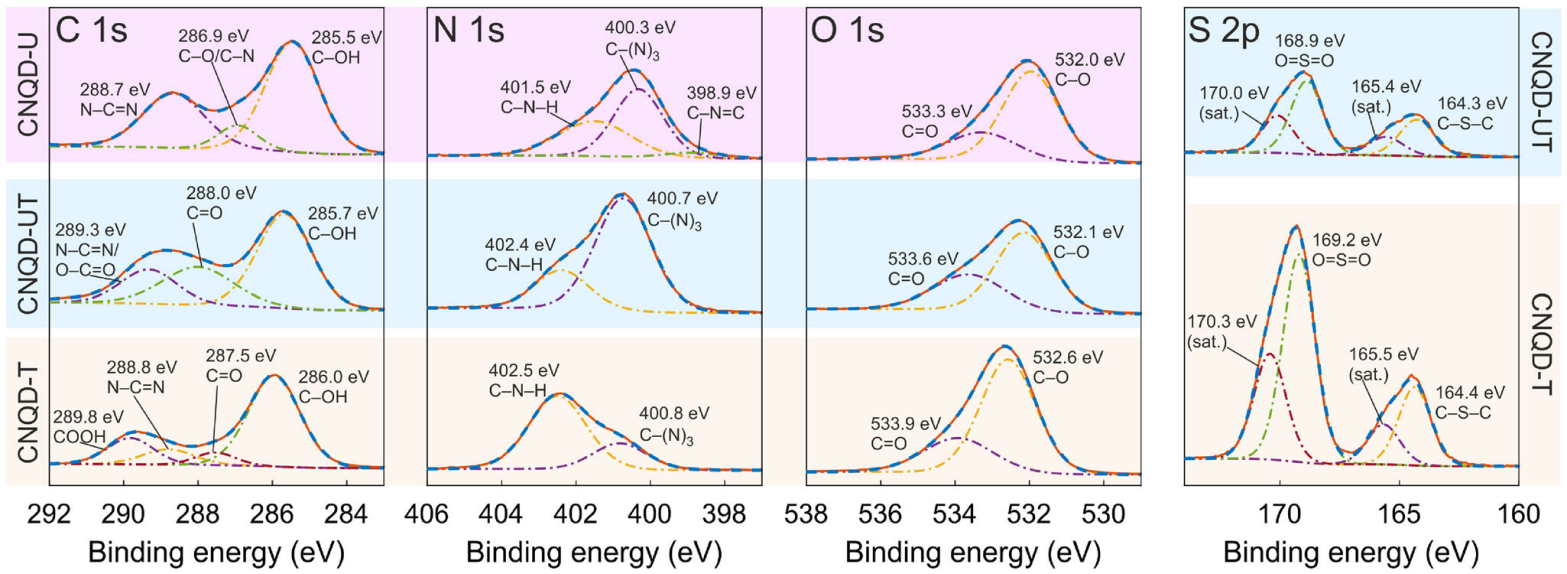
C 1s, N 1s, O 1s, and S 2p high‐resolution XPS spectra for CNQD‐U, CNQD‐UT, and CNQD‐T. Solid red lines represent the measured spectra, and dashed blue lines show the fitted envelopes. Dash‐dotted lines represent the individual fitted peaks.

High‐resolution XPS spectra of the synthesized CNQD samples are presented in Figure 2. As expected, all samples exhibited C 1s, N 1s, and O 1s peaks, while sulfur‐doped samples CNQD‐UT and CNQD‐T additionally showed S 2p signals. The corresponding elemental compositions are summarized in Table 1. Sulfur contents of 3.2 at% and 8.8 at% were observed for CNQD‐UT and CNQD‐T, respectively, confirming successful sulfur incorporation during synthesis. Sulfur doping in CNQD‐T was accompanied by an almost 10 at% increase in oxygen content, indicating a higher abundance of oxygen‐containing functional groups in this sample.

**TABLE 1:** Elemental composition of the CNQD samples determined using XPS.

	Abundance (at%)		
Element	CNQD‐U	CNQD‐UT	CNQD‐T
C	55.2	50.4	38.9
N	12.0	15.4	11.1
O	32.8	31.0	41.2
S	0	3.2	8.8

The C 1s spectrum of CNQD‐U displayed a peak at 288.7 eV, attributed to N–C = N bonding [71]. The N 1s spectrum revealed peaks at 398.9 and 400.3 eV, corresponding to C–N = C and C–(N)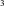 environments, respectively [72], indicating the presence of a *g*‐$C_{3}N_{4}$ core. Additional features included C 1s peaks at 285.5 eV (C–OH) [71] and 286.9 eV (C–O/C–N) [72] and an N 1s peak at 401.5 eV (C–N–H) [1, 72]. O 1s peaks at 532.0 eV (C = O) and 533.3 eV (C–O/C–OH) [75] were attributed to various oxygen‐containing groups on the surface of CNQD and to chemisorbed oxygen.

The N–C = N peak was also observed in the C 1s spectrum of CNQD‐T at 288.8 eV, while in CNQD‐UT it appeared at 289.3 eV, likely due to overlap with the O– C = O signal [1]. The N 1s spectrum of CNQD‐UT showed peaks at 400.7 and 402.4 eV, and for sample CNQD‐T these peaks were shifted upward by 0.1 eV. These peaks are tentatively assigned to C–(N)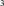 and C–N–H environments, respectively. The presence of these features suggests that the *g*‐$C_{3}N_{4}$ core is retained in sulfur‐doped CNQDs, although sulfur incorporation introduces structural defects and electron‐withdrawing effects, resulting in a substantial upward shifts in binding energies.

O 1s peaks in sulfur‐doped samples were similar to those in CNQD‐U but shifted to higher binding energies: 532.1 and 533.6 eV for CNQD‐UT, and 532.6 and 533.9 eV for CNQD‐T. These shifts are attributed to sulfur‐induced electronic effects and possible overlap with S = O signals [75]. Moreover, O–C = O functional groups present on the surface of sulfur‐doped CNQDs can undergo reduction. The C–OH peaks in the C 1s spectra were also shifted: 285.7 eV for CNQD‐UT and 286.0 eV for CNQD‐T. Additional C 1s peak assignments are provided in Figure 2. Overall, sulfur doping resulted in a consistent upward shift in C 1s, N 1s, and O 1s binding energies.

The S 2p spectrum of CNQD‐UT exhibited peaks at 164.3 and 168.9 eV (along with their satellites). The first peak could be ascribed to C–S–C units [76] and/or C–S bonds replacing N atoms, and the second peak could be attributed to O = S = O bonding [75]. CNQD‐T showed similar features but slightly shifted to higher binding energies.

**FIGURE 3:**
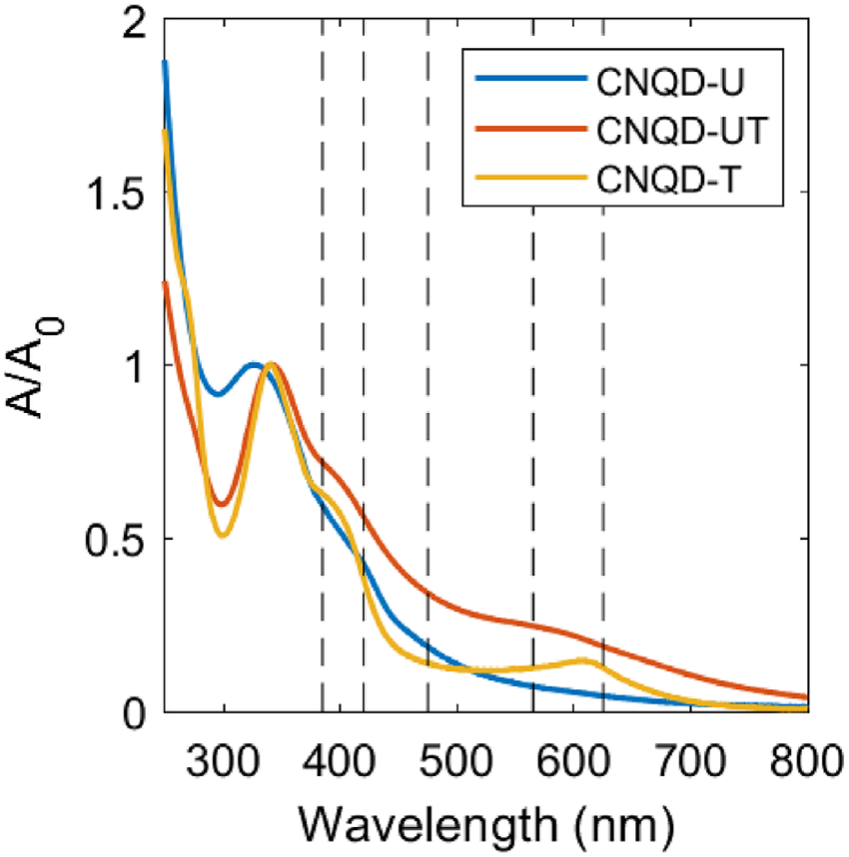
Normalized UV–vis absorption spectra of CNQD‐U (blue), CNQD‐UT (red), and CNQD‐T (yellow) in PBS at a concentration of 50 μg ${\mathrm{mL}}^{-1}$. Vertical dashed lines indicate the excitation wavelengths used in the photochemical experiments.

The UV–vis absorption spectra of the CNQD samples are presented in Figure 3. All spectra exhibited multiple absorption bands superimposed on a light scattering background. A shoulder in the 250–300 nm range was observed for all samples, corresponding to the $\pi \to \pi^{*}$ transition in the *g*‐$C_{3}N_{4}$ framework [73]. The main absorption peak appeared at 325 nm for CNQD‐U and was redshifted to 341 nm in the sulfur‐doped samples, CNQD‐UT and CNQD‐T. This peak is attributed to the $n\to \pi^{*}$ transition in the *g*‐$C_{3}N_{4}$ core, influenced by surface functional groups [1, 73]. The red shift observed in the sulfur‐doped samples is likely attributed to modifications in the electronic structure and increased structural defectiveness introduced by sulfur atoms. All samples exhibited a shoulder between 380 and 450 nm, which is possibly associated with surface states and structural defects [74]. Additionally, CNQD‐TU displayed a broad shoulder extending from 450 to 700 nm, while CNQD‐T showed a distinct peak at 610 nm. These long‐wavelength absorption features were tentatively attributed to electronic effects associated with sulfur doping.

**FIGURE 4:**
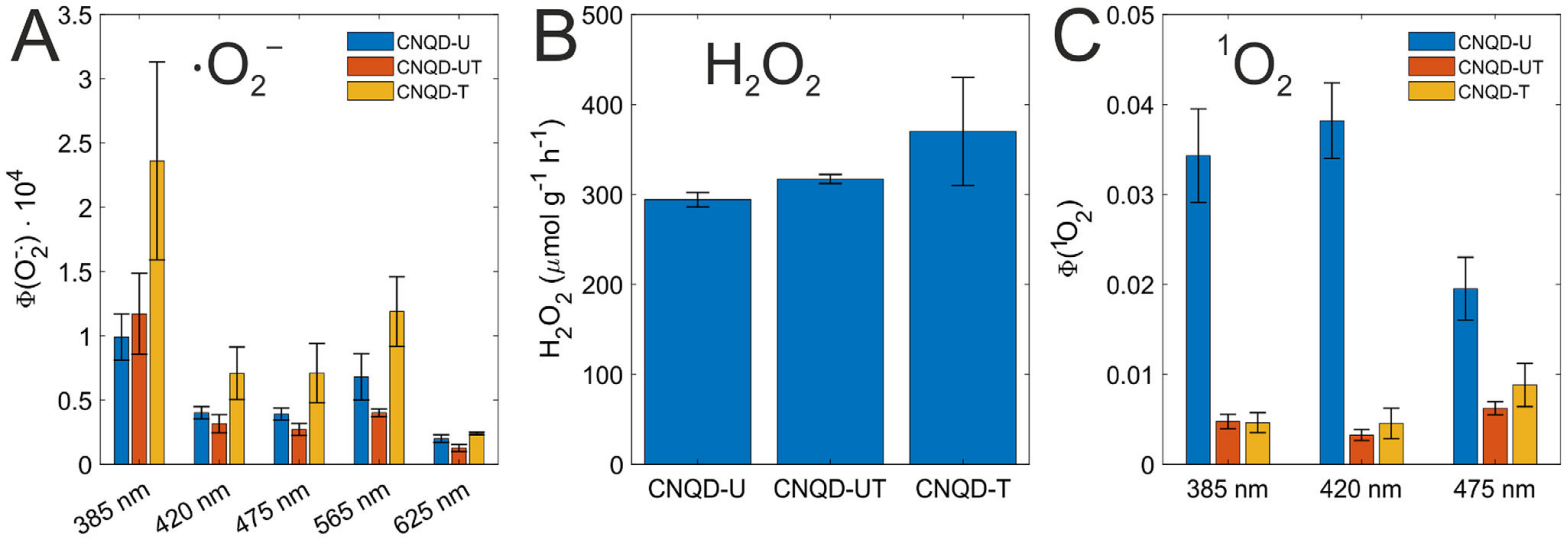
Quantification of ROS photogenerated by CNQD samples. (A) $O_{2}^{\cdot -}$ quantum yields under different excitation wavelengths, measured using cyt *c* (${\mathrm{Fe}}^{3+}$) reduction. The data were calculated based on the SOD‐inhibitable fraction of cyt *c* (${\mathrm{Fe}}^{3+}$) reduction (comparison with control data is shown in Figure S1). (B) $H_{2}O_{2}$ production under combined white (6500 K) and violet (405 nm) LED illumination, quantified using the DPD–POD assay. (C) 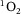 quantum yields under different excitation wavelengths, determined using SOSG. The data was calculated based on the ${\mathrm{NaN}}_{3}$‐inhibitable fraction of SOSG fluorescence (comparison with control data is shown in Figure S3). Panels A and C display slopes obtained from linear regression analysis (each based on six data points), with error bars representing 95% confidence intervals. Panel B shows mean values from duplicate measurements, with error bars indicating the observed minimum and maximum values.

Photoinduced generation of various ROS was subsequently investigated, beginning with quantification of $O_{2}^{\cdot -}$ production. This was assessed via the reduction of ferricytochrome *c* (cyt *c* (${\mathrm{Fe}}^{3+}$)) [77] under excitation at five wavelengths: 385, 420, 475, 565, and 625 nm (these are indicated as vertical dashed lines in Figure 3). Superoxide dismutase (SOD) was used in control experiments as an $O_{2}^{\cdot -}$ quencher [77]. All CNQD samples exhibited $O_{2}^{\cdot -}$ generation under each excitation condition (Figure 4A), with quantum yields ranging from $1\times 10^{-5}$ to $2\times 10^{-4}$. The highest yields were observed under 385 nm excitation. Among the samples, CNQD‐T demonstrated the greatest efficiency, producing approximately twice as much superoxide as the other CNQDs.

**FIGURE 5:**
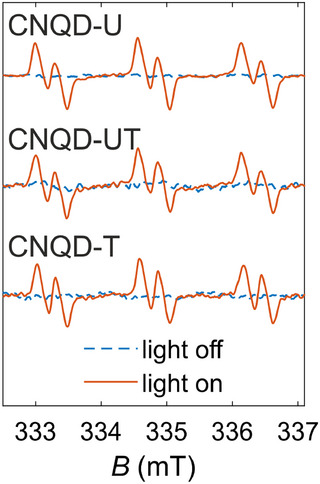
Normalized EPR spectra for CNQD‐U, CNQD‐UT, and CNQD‐T in the presence of 50 mM POBN and 5 vol% EtOH before (blue dashed lines) and after (red solid lines) irradiation.

For comparison, reported superoxide quantum yields for water‐soluble quinones span from $3\times 10^{-5}$ to $1\times 10^{-1}$ [52], while those for the endogenous coenzymes NADH and NADPH range between $2\times 10^{-9}$ and $2\times 10^{-7}$, depending on excitation wavelength [53]. Although data for other compounds are limited, the quantum yields observed for CNQDs are 3–5 orders of magnitude higher than those of NADH and NADPH, suggesting that photoexcited CNQDs could significantly elevate oxidative stress levels in biological systems.

The observed dependence of quantum yield on excitation wavelength implies that multiple types of electronic states–associated with the *g*‐$C_{3}N_{4}$ core, surface functional groups, structural defects, and heteroatoms–contribute to photocatalytic $O_{2}$ reduction. Notably, CNQD‐T, which contains 8.8 at% sulfur, exhibited enhanced $O_{2}^{\cdot -}$ production.

Interestingly, the reduction of cyt *c* (${\mathrm{Fe}}^{3+}$) by CNQD‐U was fully inhibited by SOD, confirming $O_{2}^{\cdot -}$ as the sole reducing agent. In contrast, the sulfur‐doped samples showed only partial inhibition by SOD: ∼90% for CNQD‐UT and ∼60% for CNQD‐T (Figure S1). This suggests that additional radical intermediates, beyond $O_{2}^{\cdot -}$, might be generated by the sulfur‐doped CNQDs, potentially contributing to oxygen‐independent cytotoxic effects.

Next, photoinduced $H_{2}O_{2}$ production was studied. To simulate conditions relevant to energy‐related photocatalytic applications, combined illumination with white (6500 K) and violet (405 nm) LEDs was employed. $H_{2}O_{2}$ generation was quantified using the *N*,*N*‐diethyl‐*p*‐phenylenediamine (DPD)–horseradish peroxide (POD) assay after 2 h of irradiation. The measured production rates were 294±8 μmol $g^{-1}$ $h^{-1}$ for CNQD‐U, 317±5 μmol $g^{-1}$ $h^{-1}$ for CNQD‐UT, and 370±60 μmol $g^{-1}$ $h^{-1}$ for CNQD‐T (Figure 4B), which is one order of magnitude higher than the activity of previously described cyano‐modified CNQDs [65]. The differences in $H_{2}O_{2}$ generation between the studied CNQD samples were not statistically significant (p value > 0.1)

For comparison, photocatalytic $H_{2}O_{2}$ production by bulk *g*‐$C_{3}N_{4}$ has been reported at 16.5 μmol $g^{-1}$ $h^{-1}$, increasing to 342.2 μmol $g^{-1}$ $h^{-1}$ for Ni‐$C_{3}N_{4}$ sample [78], and cobalt oxide‐decorated $C_{3}N_{4}$ demonstrated $H_{2}O_{2}$ production with a rate of 244.8 μmol $g^{-1}$ $h^{-1}$[79]. These results suggest that CNQDs represent a viable alternative to bulk *g*‐$C_{3}N_{4}$ for photocatalytic $H_{2}O_{2}$ production. The relatively high efficiency of CNQDs is likely due to their small size, which enhances the accessibility of catalytic sites, and the presence of redox‐active surface and defect states that facilitate charge transfer.

Quantum yields of 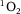 generation were measured with Singlet Oxygen Sensor Green (SOSG) as a fluorogenic probe [80] under excitation at 385, 420, and 475 nm. Excitation at longer wavelengths (565 and 625 nm) resulted in variations in SOSG fluorescence that were too weak for reliable quantification of 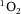 production. ${\mathrm{NaN}}_{3}$ was used in control experiments as an 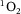 quencher [81]. 5,10,15,20‐tetrakis(*N*‐methyl‐4‐pyridyl)‐21*H*,23*H*‐porphine (${\mathrm{TMPyP}}^{4+}$), a compound with known $\Phi {(}^{1}O_{2})=0.77\pm 0.04$ [82], was used to convert the measured SOSG fluorescence intensity to the amount of the generated 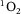 (Figure S2). Among the samples, CNQD‐U exhibited the highest quantum yields, ranging from 0.02 to 0.04 depending on the excitation wavelength (Figure 4C; Figure S3). In contrast, the quantum yields for CNQD‐UT and CNQD‐T were approximately an order of magnitude lower. The relatively low 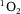 quantum yields observed for CNQDs are notable when compared to highly efficient singlet oxygen generators such as porphyrins [51, 82] and BODIPY derivatives [47], which can exhibit quantum yields between 0.1 and 0.8 in water.

Interestingly, sulfur doping appears to enhance $O_{2}^{\cdot -}$ production by CNQDs while simultaneously suppressing 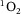 generation. This behavior contrasts with that of molecular PSs, where sulfur substitution has been shown to increase 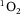 yields [20, 66].

Introducing dopants such as sulfur can alter both the electronic and surface structures of CNQDs. In addition, sulfur doping likely enhances the formation of oxygen‐containing surface groups, as indicated by the elemental composition of the CNQD samples (Table 1). These modifications may improve charge separation and transfer and increase the adsorption capacity for $O_{2}$ molecules [11, 83], thereby explaining the improved $O_{2}^{\cdot -}$ generation observed in sulfur‐doped CNQDs. Since sulfur and oxygen contents increase simultaneously in CNQD‐T, the observed rise in superoxide yield may be attributed either to sulfur doping itself or to the concomitant enrichment in oxygen‐containing surface groups. The presence of such groups can have an opposite effect on 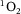 production. For instance, hydroxyl groups may act as quenchers of singlet oxygen [84].

Finally, ${\mathrm{OH}}^{.}$ generation was assessed by EPR spectroscopy using α‐(4‐Pyridyl *N*‐oxide)‐*N*‐*tert*‐butylnitrone (POBN) as a spin trap and EtOH to convert hydroxyl radicals to 1‐hydroxyethyl radical before trapping [85, 86]. No EPR signal was detected in the dark for any of the CNQD samples. Upon irradiation, all samples exhibited similar EPR spectra characterized by three doublets (Figure 5). The hyperfine splitting constants were $a_{N}=15.7$ G and $a_{H}=3.0$ G, with slight variations observed among the samples. These values align with literature data for the POBN adduct with 1‐hydroxyethyl radical [87], which forms via EtOH oxidation by hydroxyl radicals. These results confirm that all CNQD samples generate ${\mathrm{OH}}^{.}$ upon photoactivation. The hydroxyl radicals may originate either through a reductive pathway from $H_{2}O_{2}$ or an oxidative pathway from ${\mathrm{OH}}^{-}$ [88].

## 3 Conclusion

To conclude, this study demonstrates that CNQDs, both pristine and sulfur‐doped, are capable of generating multiple types of ROS upon photoactivation. For the first time, quantum yields of $O_{2}^{\cdot -}$ and 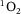 generation were quantified for CNQDs. The results reveal that sulfur doping significantly enhances $O_{2}^{\cdot -}$ production, with CNQD‐T (prepared from thiourea and citric acid) exhibiting the highest efficiency. In contrast, 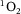 generation is substantially higher in pristine CNQDs lacking sulfur (CNQD‐U prepared from urea and citric acid). All CNQD samples also produced $H_{2}O_{2}$ and ${\mathrm{OH}}^{.}$.

The ability to produce several types of ROS, especially those associated with the Type I mechanism, is particularly promising for PDT, as it may help overcome the limitations posed by hypoxic tumor environments. Moreover, the ability of CNQDs to generate a broad spectrum of ROS makes them highly attractive for environmental applications, including pollutant degradation and wastewater treatment. Importantly, the ability to modulate the type of reactive oxygen species through sulfur doping offers a powerful strategy for precisely influencing intracellular processes or directing specific oxidation reactions.

Future work will focus on studying the interactions of CNQDs with biological systems, such as their dark and light‐induced cytotoxicity and intracellular localization.

## 4 Experimental Section

### 4.1 Materials

Urea (>99.5%), MeOH (>99.9%), KOH (>90%), cyt *c* (${\mathrm{Fe}}^{3+}$) from equine heart (>95%), SOD from bovine erythrocytes, ${\mathrm{TMPyP}}^{4+}$ (>97%), POBN (95%), and ${\mathrm{NaN}}_{3}$ (>99%) were purchased from Merck. Thiourea (>99%) was purchased from Thermo Fisher Scientific. Citric acid (>99%) was purchased from VWR Chemicals. Phosphate‐buffered saline (PBS) with pH 7.4 was prepared from tablets (Sigma–Aldrich, P4417) and deionized (DI) water. SOSG was obtained from Lumiprobe. POD (Type II, lyophilized powder, 150–250 units ${\mathrm{mg}}^{-1}$ solid) was purchased from Sigma–Aldrich, and DPD (>98%) was purchased from TCI.

### 4.2 Preparation of CNQDs

Pristine CNQDs were synthesized via a hydrothermal method using urea and citric acid, following the procedure described in ref. [73] with modifications. Sulfur‐doped CNQDs were obtained by replacing half or all of the urea with an equimolar amount of thiourea. The specific reagent quantities used in each synthesis are listed in Table 2.

**TABLE 2:** Quantities of reagents used for the preparation of different CNQD samples.

Sample	Reagents
CNQD‐U	162.0 mg (2.70 mmol) urea + 31.5 mg (0.16 mmol) citric acid
CNQD‐UT	81.0 mg (1.35 mmol) urea + 102.8 mg (1.35 mmol) thiourea + 31.5 mg (0.16 mmol) citric acid
CNQD‐T	205.5 mg (2.70 mmol) thiourea + 31.5 mg (0.16 mmol) citric acid

The reagents were dissolved in 40 mL of DI water and transferred to a 100 mL Teflon‐lined stainless steel autoclave. The mixture underwent hydrothermal treatment at 180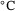 for 3 h, with a controlled heating rate of 2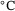 $\min^{-1}$. The resulting suspensions were dialyzed using Spectra/Por 6 membranes (10 kDa molecular weight cutoff), then sequentially filtered through syringe filters (Corning, 0.2 μm pore size, polyethersulfone membrane) and mixed cellulose ester membrane filters (MF‐Millipore, 50 nm pore size). The filtered suspensions were dried to determine solid content and subsequently redispersed in DI water at a concentration of 1 mg ${\mathrm{mL}}^{-1}$. Samples for spectroscopic and photochemical analyses were prepared by diluting these stock suspensions in PBS or DI water, as appropriate.

### 4.3 Characterization Techniques

The prepared CNQD samples were deposited on holey carbon‐supported copper grids (Electron Microscopy Sciences, HC300‐CU) by dipping the grids in undiluted suspensions of CNQDs and allowing them to dry at room temperature. TEM imaging was performed using a JEOL JEM‐2100F microscope at an accelerating voltage of 200 kV. Three TEM micrographs of each samples, each containing at least 30 particles, were analyzed to establish particle size distributions.

XPS spectra were acquired using a Nexsa G2 spectrometer (Thermo Fisher Scientific Inc.). Measurements were conducted with a 400 μm X‐ray spot size (Al K‐α radiation) and simultaneous operation of an electron flood gun in charge compensation mode. For all samples, low‐resolution survey spectra were initially collected (pass energy: 200 eV; step size: 1 eV; dwell time: 10 ms), followed by automatic identification of the present elements. Subsequently, selected peaks were recorded in high‐resolution mode (pass energy: 50 eV; step size: 0.1 eV; dwell time: 50 ms). These high‐resolution spectra were used for elemental quantification and chemical state analysis via peak fitting, performed using Avantage v6.9 software (Thermo Fisher Scientific Inc.). Peak fitting employed the Avantage smart background and variable peak components, each modeled as a Gaussian‐Lorentzian product mixture (30% Lorentzian).

### 4.4 Photoinduced ROS Generation

An integrated light source containing 5 LEDs (CHROLIS, Thorlabs) peaking at 385, 420, 475, 565, and 625 nm was used for photocatalytic superoxide and singlet oxygen generation. The light was coupled into a 3 mm liquid light guide and passed through a collimating adapter (SLSLLG3, Thorlabs) to achieve a collimated light beam. The middle part of the light beam (1×1 ${\mathrm{cm}}^{2}$) illuminated samples inside a 1 cm quartz cuvette at normal incidence. The irradiance at the front face of the cuvette was measured for each LED using an Ophir 3A‐P‐FS‐12 optical power sensor and was found to vary between 15 and 65 mW ${\mathrm{cm}}^{-2}$ depending on the excitation wavelength.

For $H_{2}O_{2}$ production, dual‐LED irradiation was employed. Specifically, a glacier white 6500 K LED and a 405 nm ultra‐high‐power LED (Mightex) were combined using a beam combiner. The light intensities were measured using a power meter (843‐R‐USB with 919P‐020‐12 sensor, Newport), yielding 270 mW ${\mathrm{cm}}^{-2}$ for the white LED and 180 mW ${\mathrm{cm}}^{-2}$ for the 405 nm LED (both were combined into a single light beam with an irradiance of 450 mW ${\mathrm{cm}}^{-2}$). The respective CNQD sample was diluted to a concentration of 0.1 mg ${\mathrm{mL}}^{-1}$ in water and then stirred (750 rpm) and irradiated in a 5 mL Schlenk flask (path length *ca*. 1 cm), which was placed in a thermostatted water bath (set to 20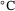) to keep the temperature constant during irradiation.

For ${\mathrm{OH}}^{.}$ generation, a LED peaking at 385 nm was used (M385L2, THORLABS). The beam was collimated with a collimator. The samples were sealed in 50 μL glass capillaries and placed 5 cm away from the collimator. The irradiance at the sample plane was measured using an Ophir 3A‐P‐FS‐12 optical power sensor and was found to be 14 mW ${\mathrm{cm}}^{-2}$.

### 4.5 Superoxide Anion Radical Detection and Quantification

$O_{2}^{\cdot -}$ generation by CNQDs was detected using cyt *c* (${\mathrm{Fe}}^{3+}$) as an electron acceptor [77]. In extracellular environments, superoxide reduces cyt *c* (${\mathrm{Fe}}^{3+}$) to cyt *c* (${\mathrm{Fe}}^{2+}$) in a 1:1 stoichiometry [89]. Samples contained 24 μM (0.3 mg ${\mathrm{mL}}^{-1}$) cyt *c* (${\mathrm{Fe}}^{3+}$). The reduction was monitored spectrophotometrically by an increase in absorbance at 550 nm, corresponding to the formation of cyt *c* (${\mathrm{Fe}}^{2+}$).

Since other reducing agents may also reduce cyt *c* (${\mathrm{Fe}}^{3+}$), control measurements were performed in the presence of 50 μg ${\mathrm{mL}}^{-1}$ SOD. The SOD‐inhibitable fraction of cyt *c* reduction was attributed specifically to superoxide. The quantum yield of superoxide generation, $\Phi (O_{2}^{\cdot -})$, was calculated as the ratio of the number of superoxide anions generated (SOD‐inhibitable fraction) to the number of photons ($N_{phot}$) absorbed by the sample using Equation (1):

(1) $$\Phi \left(O_{2}^{\cdot -}\right)=\frac{N\left(O_{2}^{\cdot -}\right)-N_{SOD}\left(O_{2}^{\cdot -}\right)}{N_{phot}}$$

where $N(O_{2}^{\cdot -})$ and $N_{SOD}\left(O_{2}^{\cdot -}\right)$ are the numbers of superoxide radicals generated in the absence and presence of SOD, respectively.

The reduction of cyt *c* (${\mathrm{Fe}}^{3+}$) is associated with a change in molar absorption coefficient at 550 nm, with $\Delta \varepsilon_{550}=21000$ $M^{-1}$ ${\mathrm{cm}}^{-1}$. The molar concentration of superoxide was calculated from the absorbance change using Equation (2):

(2) $$c\left(O_{2}^{\cdot -}\right)=\frac{\Delta A_{550}-\Delta A_{550}^{SOD}}{\Delta \varepsilon_{550}l}$$

where $\Delta A_{550}$ and $\Delta A_{550}^{SOD}$ are the absorbance changes at 550 nm in the absence and presence of SOD, respectively, and l=1 cm is the optical path length.

The number of superoxide anions was then calculated using Equation (3):

(3) $$N\left(O_{2}^{\cdot -}\right)=c\left(O_{2}^{\cdot -}\right)VN_{A}$$

where $V=1\times 10^{-3}$ L is the sample volume and $N_{A}$ is Avogadro's constant.

The number of photons absorbed by the sample was determined using Equation (4):

(4) $$N_{phot}=\frac{E\lambda }{hc}\left(1-10^{-A}\right)$$

where E is the irradiance, λ is the excitation wavelength, h is Planck's constant, c is the speed of light, and A is the absorbance at the excitation wavelength.

SOD‐inhibitable superoxide concentration was plotted against the number of absorbed photons. The slope of the resulting linear fit was taken as the quantum yield $\Phi (O_{2}^{\cdot -})$.

Absorbance spectra were recorded using a PerkinElmer Lambda 900 spectrophotometer.

### 4.6 Hydrogen Peroxide Detection and Quantification

CNQD suspensions were diluted to 0.1 mg ${\mathrm{mL}}^{-1}$ with DI water and irradiated for 2 h. Following irradiation, both irradiated and dark control samples were filtered through a polyamide membrane filter (pore size: 0.2 μm). The resulting suspensions were then diluted to 10 μg ${\mathrm{mL}}^{-1}$ by mixing 100 μL of each colloid with 900 μL of DI water.

For spectrophotometric detection of $H_{2}O_{2}$ using the DPD–POD assay, the following procedure was applied to both dark (non‐irradiated control) and irradiated samples: 1 mL of the diluted suspensions was mixed with 100 μL of a 0.25 M ${\mathrm{Na}}_{2}{\mathrm{HPO}}_{4}$ + 0.25 M ${\mathrm{NaH}}_{2}{\mathrm{PO}}_{4}$ buffer solution under continuous stirring. Subsequently, 5.7 μL of a 3.33 mg ${\mathrm{mL}}^{-1}$ DPD sulfate solution was added, followed by the rapid addition of 5.7 μL of a 0.33 mg ${\mathrm{mL}}^{-1}$ POD solution. After POD addition, the mixture was stirred for approximately 10 s to ensure uniform distribution of the reagents and immediately transferred to a quartz cuvette. UV–vis absorption spectra were recorded using a Biotek PowerWave HT spectrophotometer. Absorbance at 551 nm, corresponding to the peak absorbance of the ${\mathrm{DPD}}^{.+}$ radical cation, was recorded three times within 1 min.

The average absorbance value was converted to $H_{2}O_{2}$ concentration using a calibration curve established in the 0–0.4 mg $L^{-1}$ range of $H_{2}O_{2}$. The amount of $H_{2}O_{2}$ produced was calculated as the difference between the concentrations in the irradiated and dark samples.

### 4.7 Singlet Oxygen Detection and Quantification


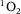
 production by CNQDs was detected using SOSG. Upon reaction with 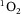, SOSG forms an endoperoxide product that exhibits fluorescence with a peak at 525 nm [80]. A 0.5 mM stock solution of SOSG was prepared in MeOH and added to PBS containing CNQDs, resulting in a final concentration of 5 μM SOSG and 1 vol% MeOH. ${\mathrm{NaN}}_{3}$, a specific quencher of 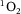 [81], was used at a concentration of 10 mM. The ${\mathrm{NaN}}_{3}$‐inhibitable increase in fluorescence intensity at 525 nm was taken as a measure of 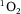 production.

The singlet oxygen quantum yield ($\Phi {(}^{1}O_{2})$) was defined as the ratio of the number of 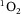 molecules generated (${\mathrm{NaN}}_{3}$‐inhibitable fraction) to the number of photons absorbed by the sample using Equation (5):

(5) $$\Phi {(}^{1}O_{2})=\frac{N{(}^{1}O_{2})-N_{{\mathrm{NaN}}_{3}}{(}^{1}O_{2})}{N_{phot}}$$

where $N{(}^{1}O_{2})$ and $N_{{\mathrm{NaN}}_{3}}{(}^{1}O_{2})$ were the numbers of singlet oxygen molecules generated in the absence and presence of ${\mathrm{NaN}}_{3}$, respectively. The number of absorbed photons, $N_{phot}$, was calculated as described in Section 4.5.

The quantum yield was estimated using a relative method, with ${\mathrm{TMPyP}}^{4+}$ in PBS as the reference compound, which has a known singlet oxygen quantum yield of $\Phi_{r}{(}^{1}O_{2})=0.77\pm 0.04$ [82]. The reference compound was photoactivated at 625 nm. Using the known $\Phi_{r}{(}^{1}O_{2})$ value, the amount of generated 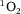 molecules was found as $N{(}^{1}O_{2})=N_{phot}\Phi_{r}{(}^{1}O_{2})$. A calibration curve was established for $N{(}^{1}O_{2})$ *vs*. $\Delta F-\Delta F_{NaN_{3}}$ (SOD‐inhibitable change in fluorescence intensity of SOSG during irradiation), as shown in Figure S2. Using the linear fit of the calibration curve, $\Delta F-\Delta F_{NaN_{3}}$ in the presence of CNQD samples was recalculated into $N{(}^{1}O_{2})$. Finally, ${\mathrm{NaN}}_{3}$‐inhibitable amount of singlet oxygen was plotted against the number of absorbed photons. The slope of the resulting linear fit was taken as the quantum yield $\Phi {(}^{1}O_{2})$.

Fluorescence spectra were recorded using an Edinburgh FLS1000 photoluminescence spectrometer under 500 nm excitation, with 1 nm excitation and emission slit band widths.

### 4.8 Hydroxyl Radical Detection

X‐band EPR spectra of trapped OH radicals were recorded using a Magnettech MiniScope MS 200 spectrometer. The following parameters were consistently applied across all measurements: microwave frequency (ν) ≈9.4 GHz, center field 3351 G, spectral width 60 G, scan time 60 s, and modulation amplitude 2000 mG. In each experiment, the CNQD concentration was 0.5 mg ${\mathrm{mL}}^{-1}$, and POBN was used as a spin trap at a concentration of 50 mM. To convert OH radicals into 1‐hydroxyethyl radicals, which were subsequently trapped by POBN, 5 vol% EtOH was added to the samples [85, 86]. The mixtures were sealed in 50 μL glass capillaries and irradiated for 3 min. EPR spectra were recorded both before and after irradiation to assess the influence of radiation.

### 4.9 Statistical Analysis

Statistical analysis was conducted using MATLAB R2023b. Superoxide and singlet oxygen quantum yields were determined by analyzing the amounts of generated species as functions of absorbed photon quantities. Each dataset consisted of six points. Linear regression was applied to each dataset, and the slopes of the fitted lines were interpreted as quantum yields. These values are shown as bars in Figure 4A,C. Error bars represent 95% confidence intervals for the estimated slopes. Measurements of photocatalytically generated $H_{2}O_{2}$ were performed in duplicate. Mean values are displayed as bars in Figure 4B, with error bars indicating the observed minimum and maximum values. Differences in $H_{2}O_{2}$ generation between samples were evaluated using two‐sample *t*‐tests.

## Conflicts of Interest

The authors declare no conflict of interest.

## Supporting information


**Supporting file**: gch270079‐sup‐0001‐SuppMat.pdf

## Data Availability

The data that support the findings of this study are openly available in Zenodo at https://doi.org/10.5281/zenodo.16875422, reference number 16875422.
