# Supplementary material for: Engineering Carbon Nitride Quantum Dots via Sulfur Doping for Controlled Reactive Oxygen Species Generation
Source: Glob Chall. 2026 Jan 8;10(1):e00447. doi: 10.1002/gch2.202500447 (PMC12783924; doi:10.1002/gch2.202500447)
Supplement: Supplementary file 1 — Supporting file: gch270079‐sup‐0001‐SuppMat.pdf [file GCH2-10-e00447-s001.pdf]

Supporting Information for:
Engineering Carbon Nitride Quantum Dots via Sulfur Doping for Controlled Reactive Oxygen Species Generation

Nikita Belko, Hanna Maltanava, Nadzeya Brezhneva, Konstantin Tamarov, Vesa-Pekka Lehto, Jani O. Moilanen, Jari T.T. Leskinen, Dmitry Semenov, Elena Filonenko, Igor Koshevoy, Jacob Schneidewind, Winnok de Vos, Polina Kuzhir

1. Additional ROS generation data

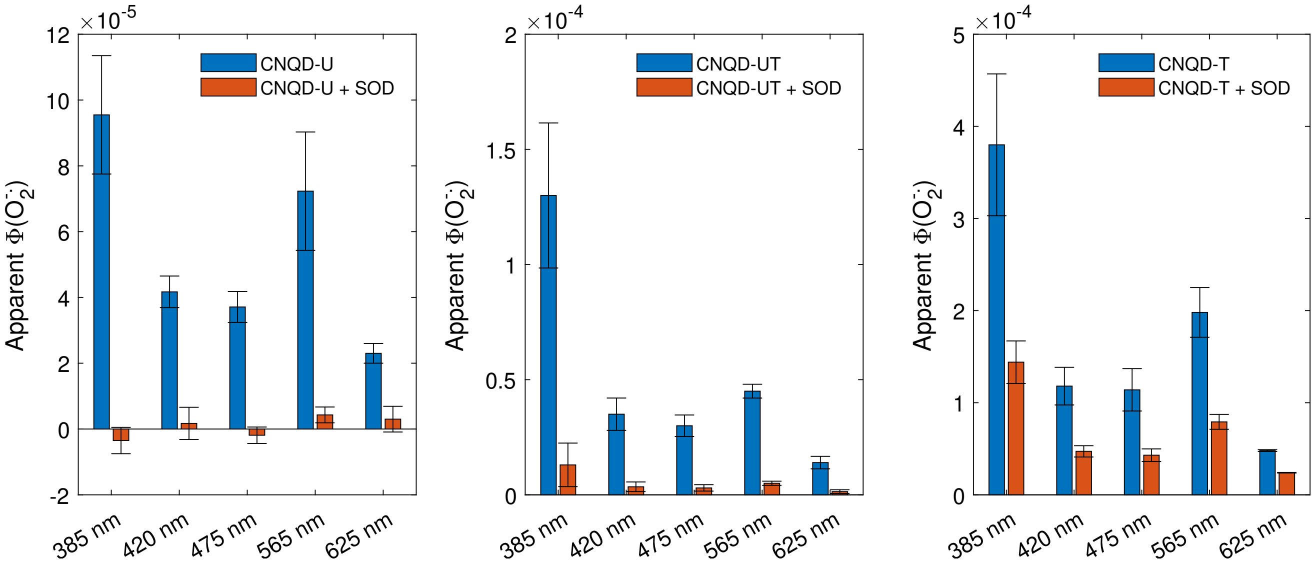

Figure S1. Apparent $O_2^{\bullet -}$ quantum yields under different excitation wavelengths, measured for different CNQD samples using cyt *c* (Fe^{3+}) reduction. The charts compare values measured without (blue bars) and with (red bars) SOD. The difference between these values, *i.e.*, the SOD-inhibitable fraction, was considered as the actual $\Phi(O_2^{\bullet -})$ and plotted in Fig. 4A of the paper.

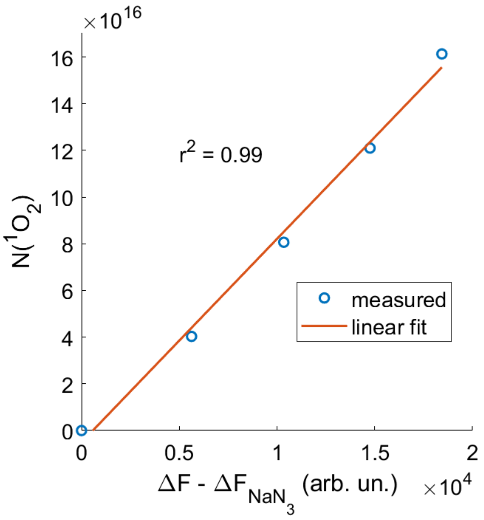

Figure S2. Dependence of the amount of the generated $^1O_2$ molecules on the increase in the SOSG fluorescence (the NaN_3-inhibitable fraction) measured using TMPyP^{4+} photoactivated at 625 nm. The amount of $^1O_2$ molecules ($N(^1O_2)$) was calculated from the number of absorbed photons (N_{phot}) using the known quantum yield of the reference compound, TMPyP^{4+} ($N(^1O_2) = N_{phot}\Phi_r\,(^1O_2)$). Blue symbols represent the measured data, while the red line corresponds to the linear fit. The linear fit was used for converting SOSG fluorescence to the amount of $^1O_2$ molecules generated by CNQDs.

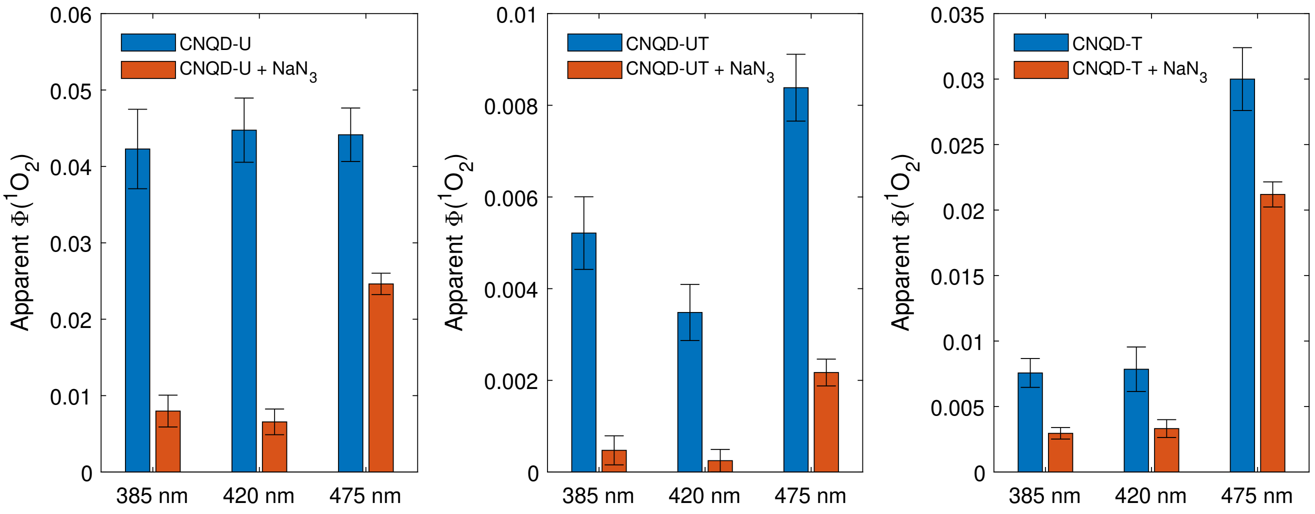

Figure S3. Apparent $^1O_2$ quantum yields under different excitation wavelengths, measured for different CNQD samples using SOSG. The charts compare values measured without (blue bars) and with (red bars) NaN_3. The difference between these values, *i.e.*, the NaN_3-inhibitable fraction, was considered as the actual $\Phi(^1O_2)$ and plotted in Fig. 4C of the paper.
